# A Prospective Evaluation of the Diagnostic Accuracy of the Point-of-Care VISITECT CD4 Advanced Disease Test in 7 Countries

**DOI:** 10.1093/infdis/jiae374

**Published:** 2024-07-24

**Authors:** Tinne Gils, Jerry Hella, Bart K M Jacobs, Bianca Sossen, Madalo Mukoka, Monde Muyoyeta, Elizabeth Nakabugo, Hung Van Nguyen, Sasiwimol Ubolyam, Aurélien Macé, Marcia Vermeulen, Sarah Nyangu, Nsala Sanjase, Mohamed Sasamalo, Huong Thi Dinh, The Anh Ngo, Weerawat Manosuthi, Supunnee Jirajariyavej, Claudia M Denkinger, Nhung Viet Nguyen, Anchalee Avihingsanon, Lydia Nakiyingi, Rita Székely, Andrew D Kerkhoff, Peter MacPherson, Graeme Meintjes, Klaus Reither, Morten Ruhwald

**Affiliations:** Department of Clinical Sciences, Institute of Tropical Medicine, Antwerp, Belgium; Global Health Institute, University of Antwerp, Wilrijk, Belgium; Ifakara Health Institute, Dar es Salaam, Tanzania; Department of Clinical Sciences, Institute of Tropical Medicine, Antwerp, Belgium; Department of Medicine, Faculty of Health Sciences, University of Cape Town, Cape Town, South Africa; Wellcome Centre for Infectious Diseases Research in Africa, Institute of Infectious Disease and Molecular Medicine, University of Cape Town, Cape Town, South Africa; Public Health Group, Malawi-Liverpool-Wellcome Programme, Blantyre, Malawi; Department of Pathology, Kamuzu University of Health Sciences, Blantyre, Malawi; Centre for Infectious Disease Research in Zambia, Lusaka, Zambia; Infectious Diseases Institute, Makerere University, Kampala, Uganda; National Lung Hospital, Ha Noi, Viet Nam; HIV Netherlands Australia Thailand Research Collaboration, Thai Red Cross AIDS Research Centre and Center of Excellence in Tuberculosis, Faculty of Medicine, Chulalongkorn University, Bangkok, Thailand; Foundation for Innovative New Diagnostics, the Global Alliance for Diagnostics, Geneva, Switzerland; Wellcome Centre for Infectious Diseases Research in Africa, Institute of Infectious Disease and Molecular Medicine, University of Cape Town, Cape Town, South Africa; Centre for Infectious Disease Research in Zambia, Lusaka, Zambia; Centre for Infectious Disease Research in Zambia, Lusaka, Zambia; Ifakara Health Institute, Dar es Salaam, Tanzania; National Lung Hospital, Ha Noi, Viet Nam; Viet Tiep Hospital, Hai Phong, Viet Nam; Bamrasnaradura Infectious Diseases Institute, Nonthaburi, Thailand; Taksin Hospital, Bangkok, Thailand; Foundation for Innovative New Diagnostics, the Global Alliance for Diagnostics, Geneva, Switzerland; Division of Infectious Disease and Tropical Medicine, Heidelberg University Hospital and Faculty of Medicine, Heidelberg University, Heidelberg, Germany; German Centre for Infection Research, Heidelberg University Hospital, Heidelberg, Germany; National Lung Hospital, Ha Noi, Viet Nam; HIV Netherlands Australia Thailand Research Collaboration, Thai Red Cross AIDS Research Centre and Center of Excellence in Tuberculosis, Faculty of Medicine, Chulalongkorn University, Bangkok, Thailand; Infectious Diseases Institute, Makerere University, Kampala, Uganda; Foundation for Innovative New Diagnostics, the Global Alliance for Diagnostics, Geneva, Switzerland; Division of HIV, Infectious Diseases and Global Medicine, Zuckerberg San Francisco General Hospital and Trauma Center, University of California, San Francisco, San Francisco, California, USA; Public Health Group, Malawi-Liverpool-Wellcome Programme, Blantyre, Malawi; School of Health and Wellbeing, University of Glasgow, Glasgow, United Kingdom; Clinical Research Department, London School of Hygiene and Tropical Medicine, London, United Kingdom; Department of Medicine, Faculty of Health Sciences, University of Cape Town, Cape Town, South Africa; Wellcome Centre for Infectious Diseases Research in Africa, Institute of Infectious Disease and Molecular Medicine, University of Cape Town, Cape Town, South Africa; Clinical Research Unit, Swiss Tropical and Public Health Institute, Allschwil, Switzerland; University of Basel, Basel, Switzerland; Foundation for Innovative New Diagnostics, the Global Alliance for Diagnostics, Geneva, Switzerland

**Keywords:** sensitivity and specificity, HIV, AIDS-related opportunistic infections, point-of-care, CD4 Antigens

## Abstract

**Background:**

CD4 measurement is pivotal in the management of advanced human immunodeficiency virus (HIV) disease. VISITECT CD4 Advanced Disease (VISITECT; AccuBio, Ltd) is an instrument-free, point-of-care, semiquantitative test allowing visual identification of CD4 ≤ 200 cells/µL or >200 cells/ µL from finger-prick or venous blood.

**Methods:**

As part of a diagnostic accuracy study of FUJIFILM SILVAMP TB LAM, people with HIV ≥18 years old were prospectively recruited in 7 countries from outpatient departments if a tuberculosis symptom was present, and from inpatient departments. Participants provided venous blood for CD4 measurement using flow cytometry (reference standard) and finger-prick blood for VISITECT (index text), performed at point-of-care. Sensitivity, specificity, and positive and negative predictive values of VISITECT to determine CD4 ≤ 200 cells/ µL were evaluated.

**Results:**

Among 1604 participants, the median flow cytometry CD4 was 367 cells/µL (interquartile range, 128–626 cells/µL) and 521 (32.5%) had CD4 ≤ 200 cells/µL. VISITECT sensitivity was 92.7% (483/521; 95% confidence interval [CI], 90.1%–94.7%) and specificity was 61.4% (665/1083; 95% CI, 58.4%–64.3%). For participants with CD4 0–100, 101–200, 201–300, 301–500, and >500 cells/µL, VISITECT misclassified 4.5% (95% CI, 2.5%–7.2%), 12.5 (95% CI, 8.0%–18.2%), 74.1% (95% CI, 67.0%–80.5%), 48.0% (95% CI, 42.5%–53.6%), and 22.6% (95% CI, 19.3%–26.3%), respectively.

**Conclusions:**

VISITECT's sensitivity, but not specificity, met the World Health Organization's minimal sensitivity and specificity threshold of 80% for point-of-care CD4 tests. VISITECT's quality needs to be assessed and its accuracy optimized. VISITECT’s utility as CD4 triage test should be investigated.

**Clinical Trials Registration**. NCT04089423.

## Teaser

CD4 measurement is essential to diagnose advanced HIV disease. In this 7-country prospective assessment, the point-of-care VISITECT CD4 Advanced Disease, performed on finger-prick blood, had an excellent specificity of 92.7% but a suboptimal specificity of 61.4%.

Determination of the CD4 cell count in people with human immunodeficiency virus (PWH) is essential for identifying advanced HIV disease (AHD) and guiding patient management. AHD is defined as CD4 < 200 cells/µL or a World Health Organization (WHO) stage 3/4 conditions in those >5 years old [1]. An estimated 630 000 died of AIDS-related illnesses in 2022 [2]. PWH with AHD are at high risk of death [3]. The proportion of people with AHD initiating antiretroviral therapy (ART) or reentering care is estimated to be up to 42% in some settings [4]. CD4 testing is an entry point to the WHO-recommended enhanced care package to reduce AHD-related mortality [5, 6]. Current guidelines recommend tuberculosis lipoarabinomannan (LAM) reflex testing in inpatients who either have tuberculosis symptoms, who have AHD or are seriously ill, or who have CD4 below 200 cells/µL. In outpatients, tuberculosis LAM screening is recommended in those who have tuberculosis symptoms, are seriously ill or at CD4 below 100 cells/µL [1, 7]. Cryptococcal antigen (CrAg) screening is indicated at CD4 below 100 cells/µL, and encouraged at CD4 below 200 cells/µL [7]. Cotrimoxazole prophylaxis is indicated at any CD4 in areas with high incidence of malaria and severe bacterial infections, and at CD4 below 350 cells/µL in other areas, but recommended at CD4 below 200 cells/µL in several countries [5, 8]. Implementation of existing point-of-care Determine TB LAM antigen assay (Determine TB LAM; Abbott) and CrAg lateral flow assay (CrAg LFA; Immy, Inc), and cotrimoxazole initiation, to reduce AHD-related mortality thus depends to a large extent on CD4 testing [9, 10].

However, CD4 test availability decreased in the global south following WHO guidance to initiate ART irrespective of CD4 count and to use viral load as the standard for treatment monitoring [11, 12]. Flow cytometry devices for CD4 count are often only found in centralized laboratories (if at all), yet baseline CD4 counts regularly become available after ART initiation, due to laboratory turn-around times [13]. In primary care settings, turn-around times are even longer [14]. This situation is unlikely to improve, as the manufacturer of widely used CD4 devices BD FACScount and FACSpresto (Becton Dickinson Biosciences) has withdrawn from the market [15]. Point-of-care CD4 devices could address some of these challenges and provide immediate results guiding patient management, but also Alere PIMA CD4 assay (PIMA; Abbott) devices are no longer produced [15]. The absence of CD4 testing has been a key barrier to scaling-up of the enhanced AHD care package [6, 16].

An instrument-free point-of-care CD4 lateral flow assay, VISITECT CD4 Advanced Disease (VISITECT; AccuBio, Ltd), allows for a visual interpretation of a CD4 count equal to or below 200 cells/µL or above 200 cells/µL. The semiquantitative test provides a result in 40 minutes and can be performed on venous or capillary blood. Within a diagnostic accuracy trial for FUJIFILM SILVAMP TB LAM (FujiLAM; Fujifilm; clinicaltrials.gov NCT04089423), all recruited participants were offered a VISITECT test on a finger-prick sample and a CD4 measurement by flow cytometry on venous blood. We compared the diagnostic accuracy of VISITECT to identify a CD4 count above or below 200 cells/µL, when performed at point-of-care, to flow cytometry as the reference standard.

## METHOD

### Study Design, Period, and Participants

This was a prospective diagnostic accuracy study, conducted in 7 countries (Malawi, South Africa, Tanzania, Thailand, Uganda, Vietnam, and Zambia) between December 2019 and December 2021, as already described elsewhere [17]. Adult (aged ≥18 years) PWH were recruited, irrespective of CD4 count or ART status, from either (1) inpatient settings irrespective of tuberculosis symptoms (inpatient group); or (2) outpatient settings if a symptom suggestive of tuberculosis (cough, night sweats, fever, or weight loss) was present (outpatient group). PWH who had received 3 or more doses of antituberculosis treatment in the last 60 days or isoniazid preventive therapy within 6 months prior to enrolment were excluded. Each country's research ethics committee approved the protocol (clinicaltrials.gov: NCT04089423). All participants provided written informed consent in their preferred language [17]. We followed the Standards for Reporting Diagnostic accuracy studies (Supplementary Material, p. 3) [18].

### Procedures

On the day of enrolment, participants underwent clinical evaluation and provided a venous blood sample for flow cytometry and a finger-prick capillary sample for VISITECT. Flow cytometry was conducted in a laboratory by a laboratory technician with WHO prequalified or equivalent devices available in the country: BD FACScount in Malawi, Tanzania, and Vietnam; BD FACSCalibur in Uganda and Thailand; Beckam Cytomics FC 500 (Beckman Coulter) in Zambia; and Beckam Aquios CL (Beckman Coulter) in South-Africa (Supplementary Table 1) [19].

Training on VISITECT procedures varied by country due to the coronavirus disease 2019 (COVID-19) pandemic, between online and onsite training, and between training by FIND master trainers, who were trained by Omega Diagnostics or local trainer-operators. Across countries, different operators performed VISITECT. In all countries, at least 1 nurse performed the test. VISITECT was also performed by medical officers in South Africa and Uganda, laboratory investigators and nurse assistants in Thaliand, field workers in Malawi; and medical officers and a research assistant in Zambia (Supplementary Table 1). Details on the study sites have been previously reported [17].

VISITECT procedures were conducted per manufacturer's instructions (Supplementary Figure 1) [20]. One operator per VISITECT test performed and interpreted the results. VISITECT operators were blinded to the results of flow cytometry and vice versa. An operator collected a capillary blood sample via finger-prick, and 30 µL was collected with a sampling device and used for the VISITECT test. The sample was added to well A by that sampling device. After 3 minutes, 1 drop of buffer was added to well A. After 17 minutes, 3 drops of buffer were added to well B. After an additional 20 minutes, results were interpreted by the operator by comparing the color intensity of the test line to a 200-μL reference line on the test device.

Operators were asked to report (1) the presence of the control line, 200-μL reference line, and test line (each 1 present/absent); (2) the comparison of color intensity (test line color stronger, the same as, or weaker [lighter] than the 200-μL reference line); and (3) the result interpretation (below the 200-μL reference line in case of the same or weaker intensity, and above the 200-μL reference line in case of darker intensity). When the control line or 200-μL reference line was absent, operators were instructed to consider the test as invalid and repeat it. Procedures for tuberculosis testing have been reported elsewhere (Supplementary Table 2) [17].

### Outcomes

The primary objective of the study was to obtain point estimates with uncertainty of the sensitivity and specificity of VISITECT to identify CD4 ≤ 200 cells/µL when compared to flow cytometry as the reference standard, overall and by patient group (inpatients or outpatients). VISITECT results were considered the results interpretated by the operator, regardless of the reported line presence or color intensity. Secondary prespecified outcomes included (1) the rate of invalid results (defined as control line or reference line reported absent) for VISITECT; and (2) the frequency and proportion of CD4 count misclassification by VISITECT, stratified by CD4 categories (0–100, 101–200, 201–300, 301–500, > 500 cells/µL) identified by cytometry. Because the overall diagnostic accuracy results showed a lower specificity for VISITECT than expected, we added post hoc estimates of the sensitivity and specificity of VISITECT to identify CD4 ≤ 200 cells/µL by ART status, composite tuberculosis reference standard result, VISITECT lot number, and country. In case of a first invalid result reported, and a repeated test, the result of the repeated test was used.

### Statistical Analysis

We described participant characteristics using median and interquartile ranges (IQR) for continuous variables and frequencies and proportions for categorical variables. The proportion of invalid VISITECT results with Clopper-Pearson 95% confidence interval (CI) was reported among participants for whom a VISITECT result was available. Point estimates with associated Clopper-Pearson 95% CI were estimated for the sensitivity, specificity, and positive and negative predictive value in participants with a VISITECT and a flow cytometry result, for all post hoc point subgroup estimates of sensitivity and specificity, and for the proportions of misclassified VISITECT test results by CD4 category. We performed 3 sensitivity analyses for diagnostic accuracy: (1) additionally excluding PWH for whom the reported VISITECT line reading indicated invalid results (control or 200-μL reference line absent), but the test result was reported as valid; (2) based on reported color intensity, regardless of result interpretation; (3) excluding 53 tests from South Africa with a lot number containing the same typographical error (these tests were assumed to be the same lot and grouped as “unknown lot” for further analysis). We compared diagnostic accuracy in subgroups by comparing 95% CIs.

Positivity of VISITECT (true positive when reference CD4 ≤ 200 cells/µL and false positive when reference CD4 > 200 cells/µL) was graphically plotted as a function of CD4 count in a generalized additive model (GAM), and stratified by subgroups. For visualization, we used the 5 most utilized lot numbers from which at least 100 tests were completed. We constructed a multivariable generalized linear (mixed) model (GLMM) with positivity of VISITECT as the outcome and log(CD4) by flow cytometry (the back-transformed logarithm of the flow cytometry CD4 results) as a predictor. Visual model comparison between the GAM and this logistic model showed that they largely overlapped (Supplementary Figure 2); however, the logistic model was preferred for modelling due to its monotonicity. Age, sex, patient group, ART status, and composite tuberculosis reference standard result were added as fixed effects, while VISITECT lot number, country, and operator (ie, individual persons, not the professional operator profile) performing VISITECT (nested within country), were considered as random-effect covariates. Adjusted odds ratios with Wald 95% CI are presented, and *P* values based on the comparison between full and reduced models, using the χ^2^ test. A *P* value of <.05 was considered statistically significant. Analyses were done with Stata (Statacorp, version 16.1) and R (version 4.3.0.) [21]. GAMs were fitted using the mgcv package and GLMMs using the lme4 package [22, 23] (Supplementary Material, p. 4f).

## RESULTS

### Participant Inclusion

**Figure 1.**
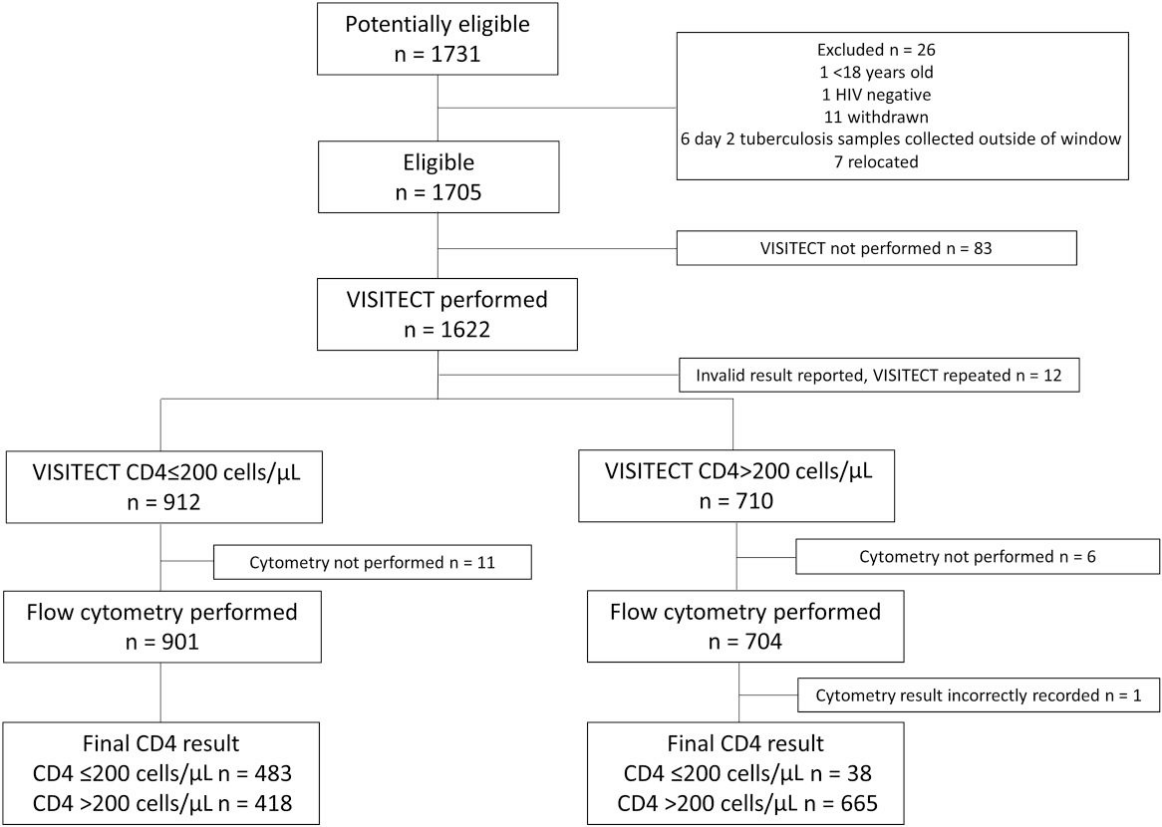
Study flow chart. In case of invalid results, the result of the repeated VISITECT was used. Abbreviation: VISITECT, Omega VISITECT CD4 Advanced Disease.

From 1731 potentially eligible participants, VISITECT results were reported for 1622 participants (Figure 1).

### Rate of Invalid Results

Among 1622 VISITECT tests, line readings were invalid in 24 cases (1.5%; 95% CI, 1.0%–2.2%). Among 1536 instances where the color intensity of the test line was reported, 28 (1.8%; 95% CI, 1.2%–2.6%) were in disagreement with the result interpretation (Supplementary Table 3).

### Participant Characteristics

Among 1622 participants with a VISITECT result, 1605 (99.0%) also had a flow cytometry result. One participant with a typographic error in the flow cytometry value was excluded. In the primary analysis, 1604 participants were included (Table 1).

**Table 1.** Participant Characteristics and Test Results

Characteristics	Value
Age, y, median (IQR)	40 (33–48)
Sex	
Female	839 (52.3)
Male	765 (47.7)
Patient group	
Inpatient	693 (43.2)
Outpatient	911 (56.8)
ART status	
On ART	1241 (77.4)
Past ART^a^	93 (5.8)
Never used ART	252 (15.7)
Unknown	18 (1.1)
Tuberculosis symptoms	
Cough	1064 (66.3)
Fever	713 (44.5)
Weight loss	1007 (62.8)
Night sweats	605 (37.7)
Seriously ill^b^	626 (39.0)
Composite tuberculosis reference standard^c^	
Positive	455 (28.4)
Negative	886 (55.2)
Unclassifiable	263 (16.4)
VISITECT result	
Above 200-μL reference line	703 (43.8)
Below 200-μL reference line	901 (56.2)
VISITECT intensity test line vs 200-μL reference line	
Stronger	700 (43.6)
The same	216 (13.5)
Weaker	620 (38.7)
Result not reported	68 (4.2)
Cytometry CD4, cells/µL, median (IQR)	367 (128–626)
Cytometry CD4 category, cells/µL	
0–100	337 (21.0)
101–200	184 (11.5)
201–300	174 (10.8)
301–500	327 (20.4)
>500	582 (36.3)

Data are No. (%) except where indicated.

Abbreviations: ART, antiretroviral therapy; IQR, interquartile range; WHO, World Health Organization; Xpert, Xpert MTB/RIF Ultra, Cepheid.

^a^Participant reported not being on ART at enrolment but having taken ART in the past.

^b^Seriously ill if any of the following present: respiratory rate > 30 breaths/min, heart rate > 120 beats/min, body mass index ≤ 18.5 kg/m^2^, systolic blood pressure < 90 mmHg, or being unable to walk unaided. Among the seriously ill, 208 (33.2%) had WHO stage 3 or 4 disease, 57.0 had stage 1 or 2 disease, and for 61 (9.7%) WHO staging data were missing.

^c^Day 1–2 sputum Mycobacteria Growth Indicator Tube or Löwenstein-Jensen culture, blood culture, Xpert urine, Xpert sputum, 2 to 3-month follow-up testing, or additional (nonstudy) testing on other samples, plus antituberculosis therapy with response.

### Overall Diagnostic Accuracy

The percentage of participants with CD4 ≤ 200 cells/µL by flow cytometry was 32.5% (95% CI, 30.2%–34.8%). The overall sensitivity of VISITECT to identify CD4 ≤ 200 cells/µL compared to flow cytometry was 92.7% (95% CI, 90.1%–94.7%), and specificity was 61.4% (95% CI, 58.4%–64.3%) (Table 2).

**Table 2.** Diagnostic Accuracy of VISITECT CD4 Advanced Disease

VISITECT CD4 Advanced Disease Result, Index Test	Flow Cytometry Result, Reference Standard			
	CD4 ≤ 200 cells/µL	CD4 > 200 cells/µL	Total	
CD4 ≤ 200 cells/µL	483	418	901	PPV, 53.6% (50.3%–56.9%)
CD4 > 200 cells/µL	38	665	703	NPV, 94.6% (92.7%–96.2%)
Total	521	1083	1604	
	Sensitivity, 92.7% (90.1%–94.7%)	Specificity, 61.4% (58.4%–64.3%)		

Sensitivity, specificity, PPV, and NPV are presented as percentages with 95% confidence intervals.

Abbreviations: NPV, negative predictive value; PPV, positive predictive value.

Among 1604 included participants, 456 (28.4%; 95% CI, 26.2%–30.7%) were misclassified by VISITECT, 418 (26.1%; 95% CI, 23.9%–28.3%) as having CD4 ≤ 200 cells/µL (median CD4 on flow cytometry 392 (IQR, 281–561), and 38 (2.4%; 95% CI, 1.7%–3.2%) as having CD4 > 200 cells/µL (median CD4 on flow cytometry 126; IQR, 63–174).

### VISITECT CD4 Results per Reference CD4 Category

**Figure 2.**
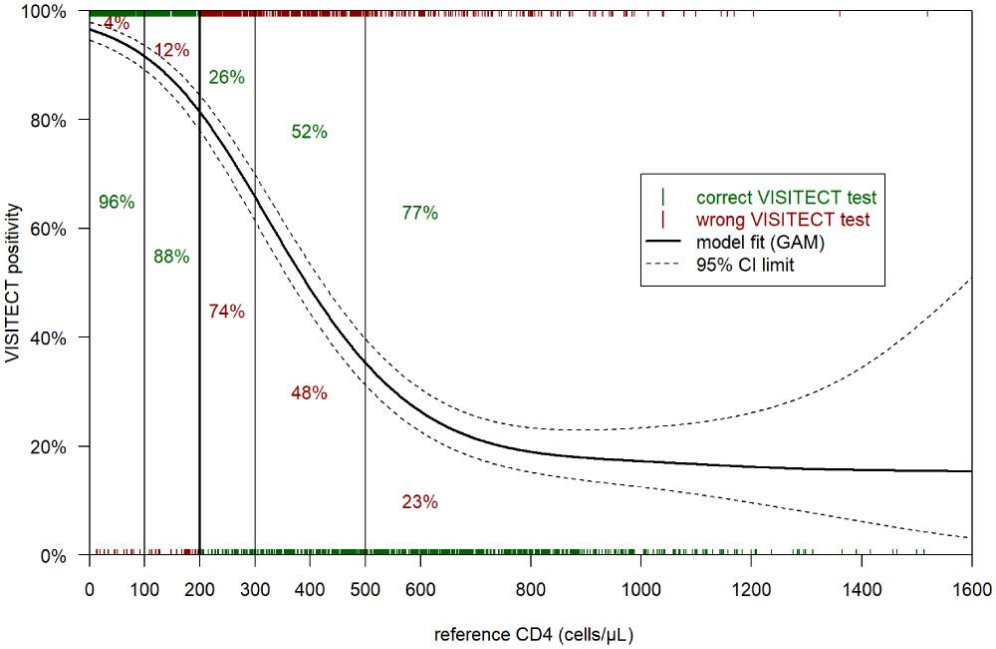
Variation in positivity of VISITECT CD4 Advanced Disease by CD4 test result by flow cytometry. The black line represents a generalized additive model of positive VISITECT results (classification as CD4 ≤ 200 cells/µL) as a function of the CD4 on flow cytometry (reference standard), with a 95% CI. This line represents true positivity when reference CD4 ≤ 200 cells/µL and false positivity when reference CD4 > 200 cells/µL. Red and green ticks represent observed results. Under 200 cells/µL, an observed result is either a true positive (100%, above, green) or false negative (0%, below, red) while under 200 cells/µL, an observed result is either a true negative (0%, below, green) or false positive (100%, above, red). Proportions present the observed proportion of correct (in green) and wrong (in red) classification by VISITECT as CD4 ≤ 200 cells/µL or >200 cells/µL, in the following CD4 categories by flow cytometry: 0–100, 101–200, 201–300, 301–500, and >500 cells/µL (empirical data). Abbreviations: CI, confidence interval; GAM, generalized additive model; VISITECT, VISITECT CD4 Advanced Disease.

Figure 2 shows the variation in positivity of VISITECT by CD4 test result, and the proportion of PWH correctly and wrongly classified by VISITECT in different CD4 categories. In the CD4 categories 0–100 and 101–200 cells/µL, VISITECT misclassified, respectively, 4.5% (95% CI, 2.5%–7.2%) and 12.5% (95% CI, 8.0%–18.2%) as CD4 > 200 cells/µL. In the categories 201–300, 301–500, and ≥501 cells/µL VISITECT misclassified, respectively, 74.1% (95% CI, 67.0%–80.5%), 48.0% (95% CI, 42.5%–53.6%), and 22.7% (95% CI, 19.3%–26.3%) as CD4 ≤ 200 cells/µL (Supplementary Table 4).

### Sensitivity Analysis

When only including 1581 participants with valid line readings, sensitivity was 93.0% (95% CI, 90.4%–95.0%) and specificity 61.3% (95% CI, 58.3%–64.2%). When including only 1536 participants with color intensity reported, sensitivity was 91.8% (95% CI, 89.0%–94.1%) and specificity 62.5% (95% CI, 59.5%–65.4%) based on that reported color intensity. When including only 1551 participants with known lot numbers, the sensitivity was 92.4% (95% CI, 89.7%–94.6%) and the specificity 60.9% (95% CI, 57.9%–63.8%) (Supplementary Table 5).

### Subgroup Analysis

**Figure 3.**
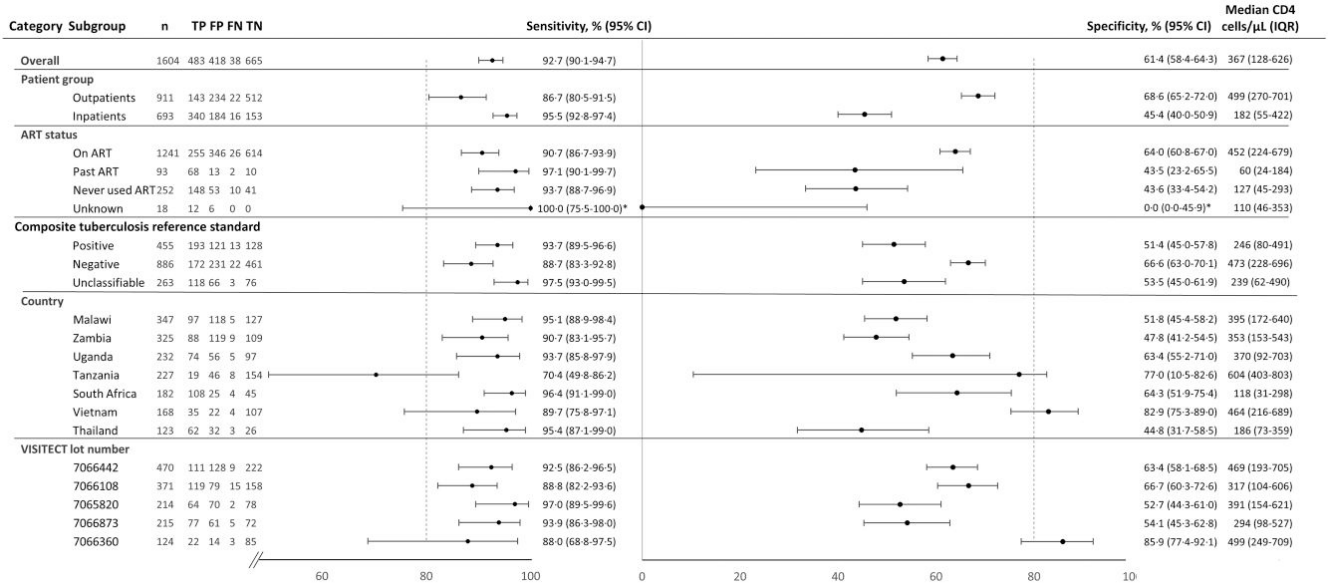
Subgroup analysis for point estimates of sensitivity and specificity of VISITECT CD4 Advanced Disease by CD4 test compared to flow cytometry. The dotted line represents the minimum/acceptable standard for sensitivity and specificity for a point-of-care CD4 test to be used as part of the enhanced care package for advanced HIV disease specified by the World Health Organization [24]. Tanzania was an outpatient-only site, South Africa an inpatient-only site. *Indicates 1-sided CI. Abbreviations: ART, antiretroviral treatment; CI, confidence interval; FN, false negative (CD4 > 200 cells/µL); FP, false positive (CD4 ≤ 200 cells/µL); IQR, interquartile range; TN, true negative (CD4 > 200 cells/µL); TP, true positive (CD4 ≤ 200 cells/µL); VISITECT, VISITECT CD4 Advanced Disease.

Results of subgroup analyses are presented in Figure 3 and Supplementary Figure 3. In a multivariable generalized linear model including log (CD4) on flow cytometry, participants who were male (vs female), who never used ART or had been on ART in the past (vs on ART), and who had a positive composite tuberculosis reference standard (vs negative) had a higher chance of having a positive VISITECT result at the same CD4 count on flow cytometry (Supplementary Table 6). VISITECT positivity at a given CD4 also varied with lot number (*P* = .003), country (*P* < .001, model without operator), and operator (*P* < .001, nested in country) (Supplementary Material, p. 4)

## DISCUSSION

In this accuracy study of VISITECT to identify advanced HIV disease, using capillary blood sampling, we found a sensitivity of 92.7% (95% CI, 90.1%–94.7%), and a specificity 61.4% (95% CI, 58.4%–64.3%) for VISITECT. Compared to the WHO minimal threshold of 80% for sensitivity and specificity for point-of-care CD4 tests to identify advanced HIV disease, VISITECT exceeded the target for sensitivity, but had lower specificity [24]. Ninety-three percent of participants with CD4 ≤ 200 cells/µL were correctly identified by VISITECT. However, the positive predictive value was low and there was important false-positive misclassification across CD4 strata, also in patients with high CD4 counts. This may lead to over testing with CrAg LFA and Determine TB LAM, and unnecessary administration of cotrimoxazole in those with CD4 counts above 350 cells/µL. Variation in VISITECT positivity at a given reference CD4 was associated with sex, ART status, composite tuberculosis reference standard result, country, VISITECT lot number, and operator. Specificity was better in subgroups with higher median CD4 counts. Due to VISITECT's acceptable sensitivity but suboptimal specificity, VISITECT may be well placed as a CD4 triage test, to rule out those with CD4 above 200 cells/µL before conducting a more specific CD4 test.

We found a similar sensitivity, but a significantly lower specificity for VISITECT compared to published studies [25, 26]. In Ndlovu et al, VISITECT sensitivity and specificity on venous blood were 95.0% (95% CI, 91.3%–97.5%) and 81.9% (95% CI, 78.2%–85.2%), and in Lechiile et al 94.1% (95% CI, 88.3%–97.6%) and 85.9% (95% CI, 83.5%–88.0%), respectively [25, 26]. On capillary blood, a 98.3% (95% CI, 95.0%–99.6%) sensitivity and a 77.2% (95% CI, 71.6%–82.2%) specificity was reported [25].

In our study, VISITECT sensitivity was in line with previous studies and above the WHO threshold [24–26]. Only 38 (2.4%) were false negatives, and 92.7% of patients would have rightly received the AHD care package [5]. Specificity of VISITECT was low; among 1084 participants with a reference CD4 > 200 cells/µL, 418 (38.6%) were misclassified as having CD4 ≤ 200 cells/µL. In published studies, false-positive misclassification after testing with venous blood was around 12%, occurring primarily in the CD4 ranges just above 200 cells/µL [25, 26]. In Ndlovu et al, the median CD4 among false positives was 252 cells/µL (IQR, 222–306 cells/µL) compared to 392 cells/µL (IQR, 281–561 cells/µL) in our study, and Lechiile et al reported that 67% (89/132) of false positives had CD4 counts below 350 cells/µL, versus 40% (165/418) in our study [25, 26]. Even those with CD4 above 500 cells/µL still had an almost 1 in 4 chance of being misclassified by VISITECT in our study. All false positives would have been unnecessarily tested with CrAg LFA, after a finger-prick or blood draw. Other participants could have received unnecessary Determine TB LAM screening, or cotrimoxazole in case of a true CD4 above 350 cells/µL (if no other indication existed). Using VISITECT results instead of cytometry thus has a potential harmful impact on patients, and the cost-effectiveness of over diagnosing CD4 ≤ 200 cells/µL should be established. However, noting its high sensitivity, VISITECT could be a considered as CD4 triage test for decentralized screening, to select candidates for a second confirmatory CD4 test. Feasibility and cost-effectiveness of this approach should be evaluated.

Variation in VISITECT diagnostic accuracy by ART and composite tuberculosis reference standard result could potentially be linked to changes in blood composition in those with tuberculosis or a high HIV load, although this assumption requires further investigation. The large CIs for VISITECT positivity in the graph of Thailand (Supplementary Figure S3) can be explained by the lower sample size (n = 123). Tanzania was the only outpatient-only site, with the highest median CD4, and less participants in the CD4 range of CD4 201–300 cells/µL. As most false-positive misclassification happened close to the 200-reference line (Figure 2), this translated into a higher subgroup specificity. A similar trend can be seen in outpatients versus inpatients, where subgroup specificity is higher in outpatients, who are generally healthier and who had a higher median CD4. We included a large variety of operator profiles, exposed to different training methods, which may explain individual operator-specific variation in VISITECT performance, reflecting the real-world rollout of this tool. In previous studies, only laboratory technicians performed VISITECT on venous blood, and nurses, doctors, and laboratory technicians performed VISITECT on capillary blood [25, 26]. VISITECT accuracy was not the primary endpoint of this trial, and VISITECT was one of many procedures. The VISITECT procedure takes 40 minutes, includes multiple different steps and difficult reading, requiring dedicated staff with repeat training [25, 27]. VISITECT result interpretation caused errors in our study; some invalid results were labelled as valid, and we observed discrepancies between reported color intensity and result interpretation in 1.8% of tests. Difficulties with VISITECT results reading has been previously reported to pose challenges when implementing an enhanced AHD care package, and repeat training and monitoring of operators may be necessary [27]. However, taking into account the discrepancies in results reported by operators in sensitivity analyses did not change our conclusions. Excluding 53 incorrectly reported lot numbers from the analysis also did not affect outcomes significantly. The lowest previously reported specificity of VISITECT (77.2%), by Ndlovu et al, was measured using finger-prick samples. In that study, operators also found finger-prick more challenging compared to venous blood sampling [25]. Bias might be introduced by sample dilution following excessive finger pressure during finger-prick sampling [25, 28]. Variation in performance of PIMA on finger-prick, and lower diagnostic accuracy after finger-prick versus venous blood with FACSpresto have been reported [29, 30]. Specific sampling technique trainings were performed, but repeat trainings and additional site visits, limited due to COVID-19, might have benefitted some operators. A situation in which CD4 testing is performed by different cadres as part of a comprehensive health assessment, and after varying levels of training intensity, reflects a situation closer to the field reality compared to previous studies.

We found multiple variables associated with VISITECT positivity in a multivariable model. Besides the hypotheses above, interaction, confounding, and multicollinearity may also influence these relationships. The effect of the true CD4 may not be completely represented by the model, and there may be unknown factors not accounted for. For instance, we did not correct for operator profiles or reference flow cytometry devices used. Therefore, it is difficult to establish a causal relationship between studied variables and VISITECT performance. What is clear from stratified analysis is that only for 2 of 7 countries (Tanzania and Vietnam) and 1 of 5 VISITECT lot numbers (with minimum 100 tests) the 95% CI for specificity included 80% [24]. This lot number (7066360) was used in 73.8% of participants from Vietnam. These 2 countries also had the highest median CD4 counts. Noting the overall large variation in subgroup performance, random variation may also contribute to this “good” performance. Graphically, we showed that despite subgroup variation, the VISITECT positivity rate remained high at high reference CD4 counts, and the curve slopes were not steep around the cutoff of 200 cells/µL, which would be necessary for an ideal test to identify this cutoff.

Strengths of our study include the large sample, with multiple settings and operators, representing the most pragmatic evaluation of VISITECT so far reported, mirroring real-life application. Yet, we performed rigorous data collection in a trial setting. We did not compare different sampling methods, which is a limitation. However, if VISITECT sampling were restricted to venous blood, requiring specific materials and training, and exposing operators to additional biohazard, this would limit VISITECT's usability in decentralized settings. We used different flow cytometry devices and did not conduct a harmonization process or external quality assessment. No interim analysis was conducted, which could have highlighted the performance challenges of VISITECT during the study.

In conclusion, in our study, VISITECT, when conducted on capillary blood, did not reach WHO threshold for specificity for a point-of-care CD4 test to be used as part of the enhanced AHD care package, by over a 10% points margin [5]. Due to the overall suboptimal performance, and considering VISITECT lot number-, operator- and country-specific variation in its diagnostic accuracy, VISITECT quality should be optimized before further use and its WHO prequalification status reevaluated. The potential for VISITECT as CD4 triage test should be further explored. Accubio should consider these results in postmarketing surveillance, as global scale-up is ongoing in less controlled conditions compared to our study [14]. New and existing manufacturers of point-of-care CD4 tests should optimize devices to meet the world's need.

## Supplementary Data

Supplementary materials are available at *The Journal of Infectious Diseases* online (http://jid.oxfordjournals.org/). Supplementary materials consist of data provided by the author that are published to benefit the reader. The posted materials are not copyedited. The contents of all supplementary data are the sole responsibility of the authors. Questions or messages regarding errors should be addressed to the author.

## Supplementary Material

jiae374_Supplementary_Data

## Notes

***Acknowledgments.*** The authors thank the study participants and their families for generously volunteering to participate in this study, as well as the study sites for their time and effort in conducting the study and assisting with the analysis of the operational data. We also thank Pamela Nabeta and Samuel Schumacher for their scientific input in study design; Agnes Malobela, Geetanjali Kataria, Sunita Singh, and Divya Soni for their study monitoring support; Rene Goliath for study support at the UCT site; and Marriott Nliwasa, Elizabeth L. Corbett, and Ailva O'Reilly at the Malawi site. Medical writing support was provided by Talya Underwood, MPhil, of Anthos Communications, Ltd, UK, funded by FIND, according to Good Publication Practice guidelines. We thank Accubio, Ltd (former Omega Diagnostics) for the donation of the VISITECT CD4 Advanced Disease tests for evaluation.

***Author contributions*.** R. S., B. S., M. Mo., J. H., H. V. N., K. R., L. N., A. D. K., G. M., and C. M. D. designed the study. R. S., B. S., M. Mo., J. H., H. V. N., S. U., T. A. N., W. M., S. J., N. V. N., A. A., K. R., L. N., A. D. K., P. M. P., G. M., C. M. D., and M. R. oversaw the study. B. S., M. Ma., M. Mo., E. N., J. H., S. U., M. V., S. N., N. S., M. S., H. T. D., L. N., P. M. P., and G. M. coordinated the individual study sites. Statistical analysis was undertaken by T. G. and B. K. J. The manuscript drafts were developed by T. G., R. S., M. R., and K. R. with input from the authors. All authors contributed to interpretation of data and editing of the article and approved the final version of the manuscript. Authors under the study consortium contributed to the individual phases of the study such as study design, coordination, and data analysis and interpretation.

***Disclaimer***. Accubio had no role in the study design, data analysis, interpretation, or preparation of this publication.

***Data sharing.*** Individual, deidentified participant data will be shared, including data dictionaries. Other documents that have been made available include the study protocol and statistical analysis plan. Templates of the informed consent forms may be shared upon request. The data will be available immediately following publication with no end date, and will be shared with anyone who wishes to access the data. The data will be available for any purpose of analyses. For data, please contact the corresponding author.

***Financial support*.** This work was supported by the Global Health Innovative Technology Fund (grant number G2017–207); the KfW Development Bank (grant number 2020 60 457); Commonwealth of Australia represented by the Department of Foreign Affairs and Trade (grant number 70957); and the Netherlands Enterprise Agency (grant number PDP15CH14). G. M. was supported by the Wellcome Trust (grant numbers 098316, 214321/Z/18/Z, and 203135/Z/16/Z), and the South African Research Chairs Initiative of the Department of Science and Technology and National Research Foundation South Africa (grant number 64787). P. M. was funded by Wellcome (206575/Z/17/Z).
