# Supplementary material for: A Prospective Evaluation of the Diagnostic Accuracy of the Point-of-Care VISITECT CD4 Advanced Disease Test in 7 Countries
Source: J Infect Dis. 2024 Jul 24;231(1):e82–90. doi: 10.1093/infdis/jiae374 (PMC11793025; doi:10.1093/infdis/jiae374)
Supplement: jiae374_Supplementary_Data [file jiae374_supplementary_data.docx]

**Supplementary file to:**

**A prospective evaluation of the diagnostic accuracy of the point-of-care VISITECT CD4 Advanced Disease test in seven countries**

**Contents**

[Supplementary material 2](#_Toc168915074)

[1. Consortium members 2](#_Toc168915075)

[2. STARD checklist 3](#_Toc168915076)

[3. R code for manuscript models and graphs 4](#_Toc168915077)

[Supplementary figures 5](#_Toc168915078)

[4. Figure S1: Standard operating procedures VISITECT CD4 Advanced Disease capillary blood 5](#_Toc168915079)

[5. Figure S2: Graphical comparison of logistic model on logarithmic scale and generealized additive model on original scale of VISITECT CD4 Advanced Disease positivity in function of CD4 on flow cytometry 5](#_Toc168915080)

[6. Supplementary Figure S3: Graphs of variation in positivity of VISITECT CD4 Advanced Disease by reference CD4 test result, stratified by country and VISITECT lot number 7](#_Toc168915082)

[Supplementary tables 8](#_Toc168915083)

[7. Table S1: Flow cytometry devices, operator profiles and training per country 8](#_Toc168915084)

[8. Table S2: List of tuberculosis tests and reference standard definitions § and participant baseline MRS and eMRS results 9](#_Toc168915085)

[9. Table S3: Overview of invalid VISITECT CD4 Advanced Disease results 10](#_Toc168915086)

[10. Table S4: CD4 count misclassification as ≤200 cells/µl and >200 cells/µl by VISITECT CD4 Advanced Disease, per CD4 category using WHO prequalified cytometers 11](#_Toc168915087)

[11. Table S5: Diagnostic accuracy of VISITECT CD4 Advanced Disease compared to WHO prequalified cytometers in sensitivity analysis 12](#_Toc168915088)

[12. Table S6: Results of univariate and multivariate generalized linear model of VISITECT CD4 Advanced Disease positivity 13](#_Toc168915089)

# Supplementary material

## Consortium members

| **First name** | **Middle name** | **Last name** | **Affiliation** |
| --- | --- | --- | --- |
| Emmanuel |  | Moreau | FIND, Geneva, Switzerland |
| Van Anh | Thi | Nguyen | FIND, Hanoi, Viet Nam |
| Andrea |  | Cavallini | FIND, Geneva, Switzerland |
| Derek | T | Armstrong | FIND, Geneva, Switzerland |
| Sergio |  | Carmona | FIND, Geneva, Switzerland |
| Tobias |  | Broger | 1. FIND, Geneva, Switzerland 2. Division of Infectious Disease and Tropical Medicine, Heidelberg University Hospital and Faculty of Medicine, Heidelberg University, Heidelberg, Germany |
| Chad | M | Centner | Division of Medical Microbiology, University of Cape Town and National Health Laboratory Service, Groote Schuur Hospital, Cape Town, South Africa |
| Minyoi |  | Maimbolwa | Centre for Infectious Diseases research in Zambia, Lusaka, Zambia |
| Brian |  | Shuma | Centre for Infectious Diseases research in Zambia, Lusaka, Zambia |
| Apichaya |  | Khlaiphuengsin | HIV-NAT, Thai Red Cross AIDS Research Centre and Excellent center in Tuberculosis, Faculty of Medicine, Chulalongkorn University, Bangkok, Thailand |
| Apicha |  | Mahanontharit | HIV-NAT, Thai Red Cross AIDS Research Centre and Excellent center in Tuberculosis, Faculty of Medicine, Chulalongkorn University, Bangkok, Thailand |
| Trang | Thi Thu | Pham | Hai Phong University of Medicine and Pharmacy, Hai Phong, Viet Nam |
| Hieu | Thi | Nguyen | Viet Tiep Hospital, Hai Phong, Viet Nam |
| Quang | Van | Nguyen | Hai Phong Lung Hospital, Hai Phong, Viet Nam |

## STARD checklist

| **STARD 2015 Checklist** | | | |  |
| --- | --- | --- | --- | --- |
|  | **Section & Topic** | **No** | **Item** | **Reported on page #** |
|  |  |  |  |  |
|  | **TITLE OR ABSTRACT** |  |  |  |
|  |  | **1** | Identification as a study of diagnostic accuracy using at least one measure of accuracy  (such as sensitivity, specificity, predictive values, or AUC) | p. 1, p. 3 |
|  | **ABSTRACT** |  |  |  |
|  |  | **2** | Structured summary of study design, methods, results, and conclusions  (for specific guidance, see STARD for Abstracts) | p. 3 |
|  | **INTRODUCTION** |  |  |  |
|  |  | **3** | Scientific and clinical background, including the intended use and clinical role of the index test | p. 7-8 |
|  |  | **4** | Study objectives and hypotheses | p. 8 |
|  | **METHODS** |  |  |  |
|  | *Study design* | **5** | Whether data collection was planned before the index test and reference standard  were performed (prospective study) or after (retrospective study) | p. 8 |
|  | *Participants* | **6** | Eligibility criteria | p. 8 |
|  |  | **7** | On what basis potentially eligible participants were identified  (such as symptoms, results from previous tests, inclusion in registry) | p. 8 |
|  |  | **8** | Where and when potentially eligible participants were identified (setting, location and dates) | p. 8 |
|  |  | **9** | Whether participants formed a consecutive, random or convenience series | p. 8 |
|  | *Test methods* | **10a** | Index test, in sufficient detail to allow replication | p. 9 |
|  |  | **10b** | Reference standard, in sufficient detail to allow replication | p. 9 |
|  |  | **11** | Rationale for choosing the reference standard (if alternatives exist) | p. 9 |
|  |  | **12a** | Definition of and rationale for test positivity cut-offs or result categories  of the index test, distinguishing pre-specified from exploratory | p. 10 |
|  |  | **12b** | Definition of and rationale for test positivity cut-offs or result categories  of the reference standard, distinguishing pre-specified from exploratory | p. 10 |
|  |  | **13a** | Whether clinical information and reference standard results were available  to the performers/readers of the index test | p. 9 |
|  |  | **13b** | Whether clinical information and index test results were available  to the assessors of the reference standard | p. 9 |
|  | *Analysis* | **14** | Methods for estimating or comparing measures of diagnostic accuracy | p. 10-11 |
|  |  | **15** | How indeterminate index test or reference standard results were handled | p. 10-11 |
|  |  | **16** | How missing data on the index test and reference standard were handled | p. 10-11 |
|  |  | **17** | Any analyses of variability in diagnostic accuracy, distinguishing pre-specified from exploratory | p. 10-11 |
|  |  | **18** | Intended sample size and how it was determined | N/A |
|  | **RESULTS** |  |  |  |
|  | *Participants* | **19** | Flow of participants, using a diagram | p. 12 |
|  |  | **20** | Baseline demographic and clinical characteristics of participants | p. 13 |
|  |  | **21a** | Distribution of severity of disease in those with the target condition | p. 13 |
|  |  | **21b** | Distribution of alternative diagnoses in those without the target condition | p. 13 |
|  |  | **22** | Time interval and any clinical interventions between index test and reference standard | p. 8 |
|  | *Test results* | **23** | Cross tabulation of the index test results (or their distribution)  by the results of the reference standard | p. 14 |
|  |  | **24** | Estimates of diagnostic accuracy and their precision (such as 95% confidence intervals) | p. 14 |
|  |  | **25** | Any adverse events from performing the index test or the reference standard | Not reported |
|  | **DISCUSSION** |  |  |  |
|  |  | **26** | Study limitations, including sources of potential bias, statistical uncertainty, and generalisability | p. 22 |
|  |  | **27** | Implications for practice, including the intended use and clinical role of the index test | p. 22 |
|  | **OTHER INFORMATION** |  |  |  |
|  |  | **28** | Registration number and name of registry | p. 3, 8 |
|  |  | **29** | Where the full study protocol can be accessed | p. 3, 8 |
|  |  | **30** | Sources of funding and other support; role of funders | p. 4, 11 |
|  |  |  |  |  |

## R code for manuscript models and graphs

The R code is added as a separate supplementary R file.

# Supplementary figures

## Figure S1: Standard operating procedures VISITECT CD4 Advanced Disease capillary blood

Reference: World Health Organization. WHO Prequalification of diagnostics. Public report: VISITECT CD4 Advanced Disease Geneva: WHO; 2022 [cited 2024 January 9]. Available from: <https://extranet.who.int/prequal/sites/default/files/whopr_files/PQDx_0384-077-00_VISTECT-CD4_AdvancedDisease_v5.0.pdf>

## Figure S2: Graphical comparison of logistic model on logarithmic scale and generealized additive model on original scale of VISITECT CD4 Advanced Disease positivity in function of CD4 on flow cytometry


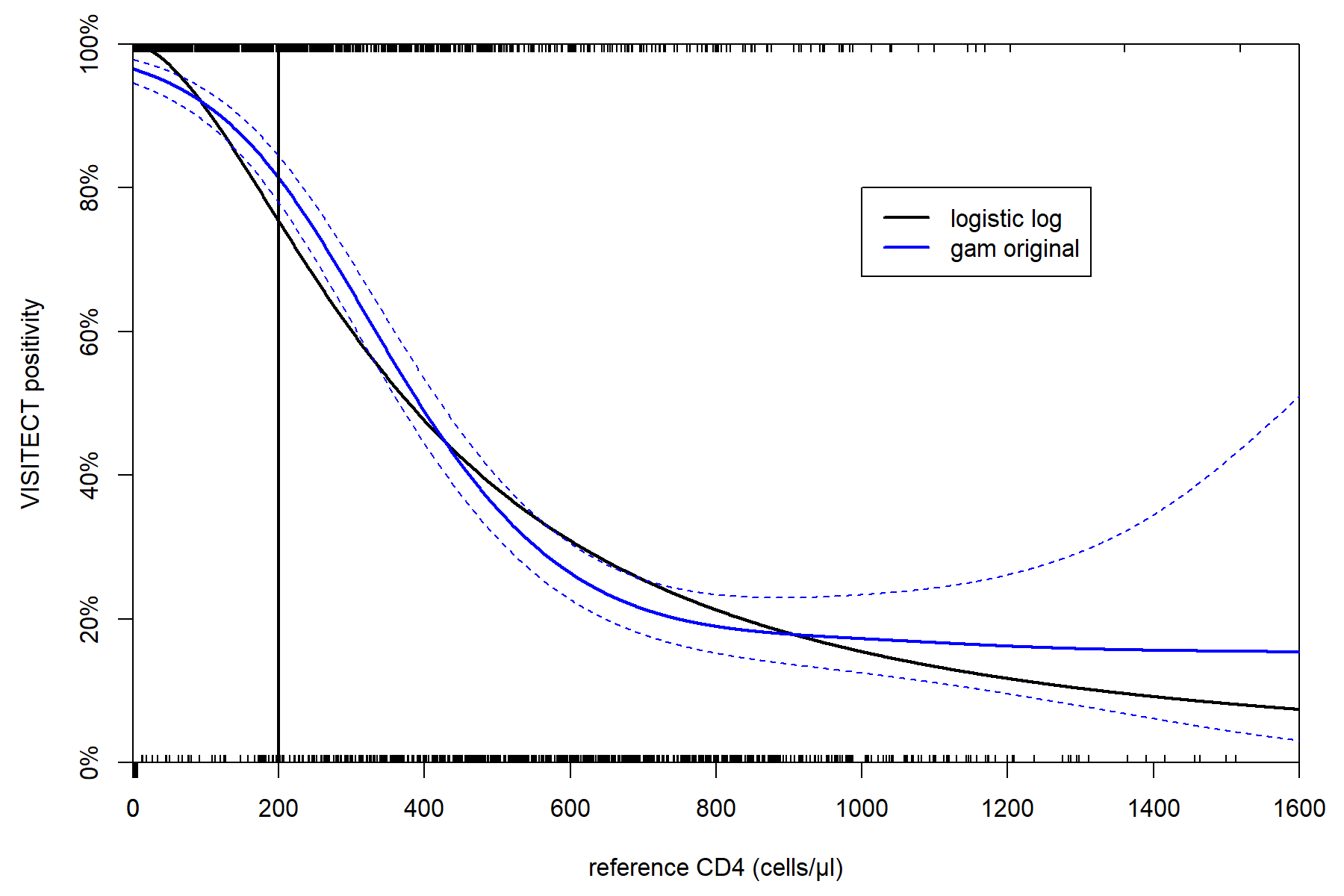


The solid black line represents a logistic model on logarithmic scale and the blue solid line a generalized additive model on original scale of positive VISITECT results (classification as CD4 ≤200 cells/µl) as a function of the CD4 on flow cytometry (reference standard), with a 95% confidence interval around the generalized additive model. These lines represents true positivity when reference CD4 ≤200 cells/µl and false positivity when reference CD4 >200 cells/µl). Under 200, an observed result is either a true positive (100%, above) or false negative (0%, below) while under 200, an observed result is either a true negative (0%, below) or false positive (100%, above).

VISITECT= VISITECT CD4 Advanced Disease

## Supplementary Figure S3: Graphs of variation in positivity of VISITECT CD4 Advanced Disease by reference CD4 test result, stratified by country and VISITECT lot number


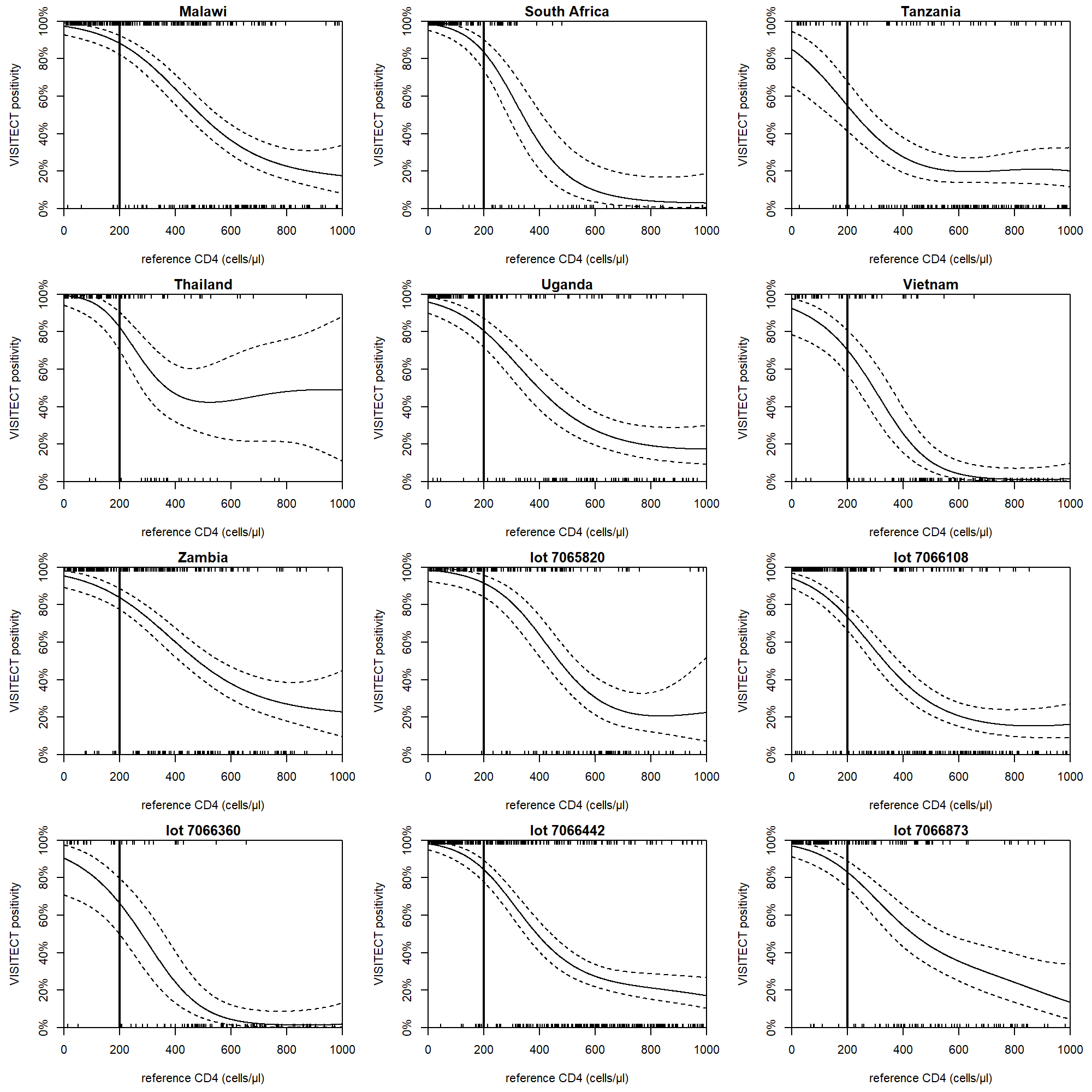


Only the 5 most utilised (n>100 tests) lot numbers (87% of tests performed) are presented.

The solid black line represents a generalized additive model of positive VISITECT results (classification as CD4 ≤200 cells/µl) as a function of the CD4 on flow cytometry (reference standard), with a 95% confidence interval. This line represents true positivity when reference CD4 ≤200 cells/µl and false positivity when reference CD4 >200 cells/µl). Ticks represent observed results. Under 200, an observed result is either a true positive (100%, above) or false negative (0%, below) while under 200, an observed result is either a true negative (0%, below) or false positive (100%, above).

VISITECT= VISITECT CD4 Advanced Disease

# Supplementary tables

## Table S1: Flow cytometry devices, operator profiles and training per country

| **Number** | **Country** | **Flow cytometry device** | **Operator** | **Operator profile** | **Training location** | **Trainer profile** |
| --- | --- | --- | --- | --- | --- | --- |
| 1 | South Africa | Beckam Aquios CL | 1 | medical officer | on site | FIND specialist |
|  |  |  | 2 | medical officer | on site | FIND specialist |
|  |  |  | 3 | nurse | on site | Local trainer |
| 2 | Malawi | BD FACScount | 1 | field worker | on site | FIND specialist |
|  |  |  | 2 | field worker | on site | FIND specialist |
|  |  |  | 3 | field worker | on site | FIND specialist |
|  |  |  | 4 | nurse | on site | FIND specialist |
|  |  |  | 5 | nurse | on site | FIND specialist |
| 3 | Zambia | Beckam Cytomics FC 500 | 1 | medical officer | on site | FIND specialist |
|  |  |  | 2 | medical officer | on site | Local trainer |
|  |  |  | 3 | medical officer | online | FIND specialist |
|  |  |  | 4 | nurse | on site | Local trainer |
|  |  |  | 5 | research assistant | on site | Local trainer |
| 4 | Uganda | BD FACSCalibur | 1 | medical officer | on site | FIND specialist |
|  |  |  | 2 | nurse | on site | FIND specialist |
|  |  |  | 3 | nurse | on site | FIND specialist |
|  |  |  | 4 | nurse | on site | FIND specialist |
| 5 | Tanzania | BD FACScount | 1 | nurse | on site | FIND specialist |
|  |  |  | 2 | nurse | on site | FIND specialist |
| 6 | Vietnam | BD FACScount | 1 | nurse | on site | Local trainer |
|  |  |  | 2 | nurse | on site | Local trainer |
| 7 | Thailand | BD FACSCalibur | 1 | lab investigator | online | FIND specialist |
|  |  |  | 2 | lab investigator | on site | Local trainer |
|  |  |  | 3 | nurse | on site | Local trainer |
|  |  |  | 4 | nurse | online | FIND specialist |
|  |  |  | 5 | nurse | on site | Local trainer |
|  |  |  | 6 | nurse assistant | on site | Local trainer |
|  |  |  | 7 | nurse assistant | on site | Local trainer |
|  |  |  | 8 | nurse assistant | on site | Local trainer |

Training on VISITECT procedures varied by country. Initially, local operators and trainer-operators were trained by FIND specialists on site, who had been trained by Omega Diagnostics (Alva, UK). Following COVID-19-related travel restrictions, online training of operator-trainers happened with slides and a quiz. Those trainer-operators then trained other operators and shared training videos for review.

## Table S2: List of tuberculosis tests and reference standard definitions § and participant baseline MRS and eMRS results

|  |  | **MRS** | **eMRS** | **CRS†** |
| --- | --- | --- | --- | --- |
| 1–2 Sputum MGIT culture* | | YES | YES | YES |
| 1–2 Sputum LJ culture* | | YES | YES | YES |
| Blood culture* | | YES | YES | YES |
| Urine Xpert Ultra | | YES | YES | YES |
| Sputum Xpert Ultra | | YES | YES | YES |
| Additional (non-study) testing** | | NO | YES | YES |
| 2–3-month follow-up testing | | YES | YES | YES |
| Anti-TB therapy with response | | NO | NO | YES |

CRS=composite reference standard, eMRS= extended microbiological reference standard, LJ= Löwenstein-Jensen, MGIT= Mycobacteria Growth Indicator Tube, MRS= microbiological reference standard, MTB= *Mycobacterium tuberculosis,* NTM= nontuberculous mycobacteria.

^*^ Including MTB complex confirmation and NTM determination

^**^ Any additional mycobacterial culture and/or Xpert/Ultra from other samples (e.g., pleural fluid, tissue biopsy, etc.) performed based on routine clinical indication.

†Chest X-Ray, AlereLAM and smear results might be considered as part of the clinical decision-making (as per country routine).

§ The respective reference standard is considered positive if any of those marked with “YES” are positive/apply. The MRS/eMRS is negative if none of the tests marked with “YES” are positive and at least one negative sputum culture is available. CRS is negative if none of those marked with “YES” are positive/apply and participant has no symptoms at 2–3-month follow-up. Unclassifiable is neither reference standard positive nor reference standard negative.

Among 1604 included participants, 264 (16.5%) tested positive, 1272 (79.3%) negative and 68 (4.2%) were unclassifiable on MRS and 270 (16.8%) were positive, 1271 (79.2%) negative and 63 (3.9%) unclassifiable on eMRS.

## Table S3: Overview of invalid VISITECT CD4 Advanced Disease results

| **Type of result** | **Total reported** | **N invalid** | **Proportion,**  **95% confidence interval** | **Reason for invalidity** |
| --- | --- | --- | --- | --- |
| Line readings | 1622 | 24* | 1.5% (1.0–2.2%) | missing control line (8), |
|  |  |  |  | missing 200-reference line (12) |
|  |  |  |  | missing control and reference line (4) |
| Colour intensity | 1536 | 28 | 1.8% (1.2–2.6%) | 14 stronger intensity than 200-reference line classified as below reference |
|  |  |  |  | 9 the same intensity as 200-reference line classified as above reference |
|  |  |  |  | 5 lighter intensity as 200-reference line classified as above reference |

*11/24 reported as invalid by operator and repeated, 13/24 interpreted as valid and not repeated.

## Table S4: CD4 count misclassification as ≤200 cells/µl and >200 cells/µl by VISITECT CD4 Advanced Disease, per CD4 category using WHO prequalified cytometers

| Flow cytometry result category in cells/µl | Total | VISITECT result  ≤200 cells/µl | VISITECT result  >200 cells/µl | Correctly classified | Wrongly classified |
| --- | --- | --- | --- | --- | --- |
| 0−100 | 337 | 322 | 15 | 95.6 (92.8-97.5) | 4.5 (2.5-7.2) |
| 101−200 | 184 | 161 | 23 | 87.5 (81.8-91.9) | 12.5 (8.0-18.2) |
|  |  |  |  | Wrongly classified | Correctly classified |
| 201−300 | 174 | 129 | 45 | 74.1 (67.0-80.5) | 25.9 (19.5-33.0) |
| 301−500 | 327 | 157 | 170 | 48.0 (42.5-53.6) | 52.0 (46.4-57.5) |
| >500 | 582 | 132 | 450 | 22.7 (19.3-26.3) | 77.3 (73.7-80.7) |
| Correctly and wrongly classified participants by VISITECT are presented as proportions with 95% confidence intervals | | | | | |

## Table S5: Diagnostic accuracy of VISITECT CD4 Advanced Disease compared to WHO prequalified cytometers in sensitivity analysis

| Category | n | TP | FP | FN | TN | Sensitivity | Specificity | CD4 |
| --- | --- | --- | --- | --- | --- | --- | --- | --- |
| Sensitivity analysis 1 | 1581 | 475 | 414 | 36 | 655 | 93.0 (90.4-95.0) | 61.3 (58.3-64.2) | 367 (130-627) |
| Sensitivity analysis 2 | 1536 | 439 | 397 | 39 | 661 | 91.8 (89.0-94.1) | 62.5 (59.5-65.4) | 384 (142-641) |
| Sensitivity analysis 3 | 1551 | 451 | 416 | 37 | 647 | 92.4 (89.7−94.6) | 60.9 (57.9−63.8) | 375 (140-633) |

Sensitivity and specificity are presented as proportions with 95% confidence intervals, CD4 are medians with interquartile ranges of CD4 on cytometry

FN= False negative (CD4>200cells/µl), FP= False positive (CD4≤200cells/µl), NPV= negative predictive value, PPV= positive predictive value, TN= True negative (CD4>200cells/µl), TP= True positive (CD4≤200cells/µl)

Sensitivity analysis 1 includes only participants for whom the control line and 200 line were reported as being present in original or repeat (in case first reading was invalid) reading and the analysis is based on reported result interpretation.

Sensitivity analysis 2 includes only participants for whom comparison of colour intensity of test line colour versus 200 line was reported and the analysis is based on those results, regardless of final reported interpretation.

Sensitivity analysis 3 includes only participants for whom the VISITECT lot number was confirmed, excluding 53 participants for which the same erroneous lot number was recorded.

| Table S6: Results of univariate and multivariate generalized linear model of VISITECT CD4 Advanced Disease positivity  \| **Fixed effects** \| **Univariate analysis** \| \| \| \| **Multivariate analysis** \| \| \| \| \| --- \| --- \| --- \| --- \| --- \| --- \| --- \| --- \| --- \| \| **Variable** \| **OR** \| **95% CI** \| \| **p-value** \| **aOR** \| **95% CI** \| \| **p-value** \| \| log(CD4), cells/µl \| 0.1726 \| 0.1414 \| 0.2083 \| <0.001 \| 0.2029 \| 0.1627 \| 0.2531 \| <0.001 \| \|  \|  \|  \|  \|  \|  \|  \|  \|  \| \| Age, years \| 0.9943 \| 0.9833 \| 1.0055 \| 0.317 \| 0.9969 \| 0.9843 \| 1.0096 \| 0.628 \| \| Sex male (vs female) \| 1.0992 \| 0.8565 \| 1.4089 \| 0.456 \| 1.3709 \| 1.0210 \| 1.8406 \| 0.037 \| \| Inpatient (vs outpatient) \| 2.0513 \| 1.5785 \| 2.6676 \| <0.001 \| 1.4579 \| 0.9184 \| 2.3141 \| 0.113 \| \| ART: past ART (vs on ART) \| 1.9999 \| 0.9435 \| 4.4612 \| <0.001 \| 2.2620 \| 1.0091 \| 5.0708 \| 0.004 \| \| ART: don't know (vs on ART) \| Inf \| 0.0000 \| Inf \| Inf \| 0.0000 \| Inf \| \| ART: never used (vs on ART) \| 1.3998 \| 0.9415 \| 2.0956 \| 1.3614 \| 0.8602 \| 2.1547 \| \| Composite TB reference standard: Positive (vs. Negative) \| 1.5568 \| 1.1625 \| 2.0855 \| 0.002 \| 1.5880 \| 1.1370 \| 2.2179 \| 0.011 \| \| Composite TB reference standard: Unclassifiable (vs. Negative) \| 1.6092 \| 1.1233 \| 2.3101 \| 1.5295 \| 0.9986 \| 2.3426 \| \|  \|  \|  \|  \|  \|  \|  \|  \|  \| \| **Random effects** \| **Univariate analysis** \| \| \| \| **Multivariate analysis** \| \| \| \| \| **Variable** \| **Variance** \| **SD (of Variance)** \| \| **p- value** \| **Variance** \| **SD (of Variance)** \| \| **p- value** \| \| Country* \| 0.248 \| 0.498 \| \| <0.001 \| 0.294 \| 0.542 \| \| <0.001 \| \| Lot number \| 0.608 \| 0.779 \| \| <0.001 \| 0.152 \| 0.389 \| \| 0.003 \| \| Operator† \| 0.780 \| 0.883 \| \| <0.001 \| 0.743 \| 0.862 \| \| <0.001 \| \| Note: random slopes: \|  \|  \|  \|  \|  \|  \|  \|  \| \| "Model failed to converge with max\|grad\| = 0.183029 (tol = 0.002, component 1)" \| \| \|  \|  \|  \|  \|  \|  \| \| * Note: comparison of models without operator (because operator nested within country) \| \| \| \|  \|  \|  \|  \|  \| \| † Note: comparison of models with country (because operator nested within country)  CD4 is reference CD4 on World Health Organization prequalified flow cytometry \| \| \|  \|  \|  \|  \|  \|  \|   aOR= adjusted odds ratio,ART= antiretroviral treatment, CI= confidence interval, inf= infinite, OR= odds ratio, SD= standard deviation, TB= tuberculosis |
| --- | --- | --- | --- | --- | --- | --- | --- | --- | --- | --- | --- | --- | --- | --- | --- | --- | --- | --- | --- | --- | --- | --- | --- | --- | --- | --- | --- | --- | --- | --- | --- | --- | --- | --- | --- | --- | --- | --- | --- | --- | --- | --- | --- | --- | --- | --- | --- | --- | --- | --- | --- | --- | --- | --- | --- | --- | --- | --- | --- | --- | --- | --- | --- | --- | --- | --- | --- | --- | --- | --- | --- | --- | --- | --- | --- | --- | --- | --- | --- | --- | --- | --- | --- | --- | --- | --- | --- | --- | --- | --- | --- | --- | --- | --- | --- | --- | --- | --- | --- | --- | --- | --- | --- | --- | --- | --- | --- | --- | --- | --- | --- | --- | --- | --- | --- | --- | --- | --- | --- | --- | --- | --- | --- | --- | --- | --- | --- | --- | --- | --- | --- | --- | --- | --- | --- | --- | --- | --- | --- | --- | --- | --- | --- | --- | --- | --- | --- | --- | --- | --- | --- | --- | --- | --- | --- | --- | --- | --- | --- | --- | --- | --- | --- | --- | --- | --- | --- | --- | --- | --- | --- | --- | --- | --- | --- | --- | --- | --- | --- | --- | --- | --- | --- | --- | --- | --- | --- | --- | --- | --- | --- | --- |
